# The applied value in brain gray matter nuclei of patients with early-stage Parkinson’s disease : a study based on multiple magnetic resonance imaging techniques

**DOI:** 10.1186/s13005-023-00371-4

**Published:** 2023-06-29

**Authors:** Heng Meng, Duo Zhang, Qiyuan Sun

**Affiliations:** Department of Radiology, Affiliated Hospital of BeiHua University, Jilin, 132011 China

**Keywords:** Parkinson’s disease, Magnetic resonance imaging, Diffusion tensor imaging, Diffusion kurtosis imaging, Quantitative susceptibility mapping, Susceptibility weighted imaging

## Abstract

**Purpose:**

This study compares the observation efficiency of brain gray matter nuclei of patients with early-stage Parkinson’s disease among various Magnetic Resonance Imaging techniques, which include susceptibility weighted imaging (SWI), quantitative susceptibility imaging (QSM), diffusion tensor imaging (DTI) and diffusion kurtosis imaging (DKI). Based on the findings, this study suggests an efficient combination of scanning techniques for brain gray matter nuclei observation, aiming to provide an opportunity to advance the understanding of clinical diagnosis of early-stage Parkinson’s disease.

**Methods:**

Forty examinees, including twenty patients who were clinically diagnosed with early Parkinson’s disease with a course of 0.5-6 years (PD group) and twenty healthy controls (HC group), underwent head MRI examination. Philips 3.0T (tesla) MR machine was used to measure the imaging indexes of gray matter nuclei in patients with early Parkinson’s disease. SWI, QSM, DTI and DKI were used for diagnosis. SPSS (Statistical Product and Service Solutions) 21.0 was used for data analysis.

**Results:**

When SWI was used, fifteen PD patients and six healthy volunteers were diagnosed correctly. The sensitivity, specificity, positive predictive value, negative predictive value and diagnostic coincidence rate about the diagnosis of nigrosome-1 on imaging were 75.0%, 30.0%, 51.7%, 54.5% and 52.5% respectively. By contrast, when QSM was used, 19 PD patients and 11 healthy volunteers were diagnosed correctly. The sensitivity, specificity, positive predictive value, negative predictive value and diagnostic coincidence rate about the diagnosis of Nigrosome-one on imaging were 95.0%, 55.0%, 67.9%, 91.7% and 75.0% respectively. The mean kurtosis (MK) value within both the substantia nigra and thalamus, together with the mean diffusivity (MD) within both the substantia nigra and the head of caudate nucleus in PD group was greater than that of HC group. The susceptibility values within the substantia nigra, red nucleus, head of caudate nucleus and putamen of PD group was greater than that of HC group. The MD value in substantia nigra reveals the optimal diagnostic efficiency to distinguish the HC group and the PD group, followed by the MK value in substantia nigra. Specifically, the maximum area under ROC curve (AUC) of the MD value was 0.823, the sensitivity 70.0%, the specificity 85.0%, and the diagnostic threshold 0.414. The area under ROC curve (AUC) of the MK value was 0.695, the sensitivity 95.0%, the specificity 50.0%, and the diagnostic threshold was 0.667. Both of them were statistically significant.

**Conclusions:**

In the early diagnosis of Parkinson’s disease, QSM is more efficient than SWI in observing nigrosome-1 in substantia nigra. In the early diagnosis of Parkinson’s disease, MD and MK values of substantia nigra in DKI parameters have higher diagnostic efficiency. The combined scanning of DKI and QSM has the highest diagnostic efficiency and provides imaging basis for clinical diagnosis of early Parkinson’s disease.

## Background

Parkinson’s disease (PD), also known as paresis agitans, is a common extrapyramidal disease in the elderly. It is only second to Alzheimer’s disease in terms of incidence among all the brain degenerative diseases. At present, the etiology and pathogenesis of Parkinson’s disease are not clear. Modern medical research suggests that the etiology of Parkinson’s disease may include age and heredity, and the pathogenesis includes oxidative stress injury, excitatory neurotoxicity, homocysteine, immune inflammatory reaction, mitochondrial dysfunction, apoptosis and autophagy [1]. Disorder of iron metabolism and abnormal deposition in brain may lead to neurodegenerative diseases such as Parkinson’s disease. Studies have shown that the iron content in substantia nigra of patients with Parkinson’s disease increased significantly [2], and with the aggravation of the disease, the iron deposition increased [3]. Although the biological mechanism of abnormal iron metabolism in patients with Parkinson’s disease is not clear, it is certain that the disease is closely related to the imbalance of iron absorption, storage and release in vivo, which is likely to be the main factor leading to the loss of dopamine neurons and abnormal aggregation of α-synuclein [4].

In the conventional plain scan sequence, the substantia nigra of normal people showed high signal on T1 weighted imaging (T1WI) and proton density weighted imaging (PDWI), but low signal on T2 weighted imaging (T2WI) and diffusion tensor imaging (DTI) [5]. In patients with Parkinson’s disease, the outline and volume of substantia nigra were abnormal, and the dense part of substantia nigra atrophied and narrowed. Some studies have found that on T1WI, the substantia nigra signal of PD patients can be reduced with the progress of disease [6]. T2*WI、SWI and other techniques are more precise and accurate in displaying the anatomical structure of substantia nigra [7]. Some studies have shown that high signal circular area can be seen in substantia nigra on T2*WI, which is confirmed to be corresponding to substantia nigra-1, which is the most vulnerable site of Parkinson’s disease [8]. Because diffusion tensor imaging (DTI) is the only noninvasive examination method to observe and track the white matter fiber tracts based on the water molecule moving direction, it can show the changes of microstructure before the macroscopic changes. Some researchers found that the FA (fractional anisotropy) values of substantia nigra, striatum, putamen and other nuclei in Parkinson’s disease patients were significantly decreased, suggesting nuclear degeneration [9]. However, some studies found that FA value and MD (mean diffusivity) value of PD patients had no significant change compared with those of normal control group [10]. Schwarz et al. conducted a meta-analysis on the previous literature and found that FA and MD values were not credible as diagnostic indicators [11]. Therefore, the reliability of DTI in the diagnosis of PD needs further study and analysis. DKI is an extension of diffusion tensor imaging (DTI). In the study of Parkinson’s disease with DKI technique, it was found that the MK values of substantia nigra, caudate nucleus, putamen and globus pallidus of PD patients were significantly higher than those of normal people, and FA values of substantia nigra were significantly higher than those of normal people. FA values of other gray matter nuclei were not statistically different from those of normal people. The common parameters of DTI included MD (mean diffusivity), AD (axial diffusivity), RD (radial diffusivity) and ADC (average diffusion coefficient). There was no significant difference between Parkinson’s disease patients and normal control group [12].

Therefore, the study compared the DKI-MK (mean kurtosis) value, DKI-AK (axial kurtosis) value, DKI-RK (radial kurtosis) value, DKI-RD (radial diffusivity) value, DKI-FA (fractional anisotropy) value, DTI-FA value, DTI-ADC value, magnetic susceptibility value of deep gray matter nuclei in PD group and HC group, and observed and determined the swallow tail sign of bilateral substantia nigra in SWI and QSM images, so as to provide objective basis for MRI diagnosis of Parkinson’s disease.

### Research object

The study was approved by the ethics committee of Affiliated Hospital of Beihua University. All examinees signed informed consent. Forty examinees underwent head MRI examination. Twenty patients (PD group) were clinically diagnosed with early Parkinson’s disease, 8 males and 12 females, with an average age of 66.50 ± 9.65 years and a course of 0.5-6 years. The average score of the UPDRS - III (Unified Parkinson’s Disease Rating Scale-III) was (18.4 ± 9.4). The other twenty examinees were healthy controls (HC group), 13 males and 7 females, with an average age of 61.25 ± 6.87 years.

According to the latest diagnostic criteria of PD published by the movement disorder Society (MDS) in 2015, the patients with secondary Parkinson’s syndrome were excluded; the Hoehn Yahr grading scale of PD was 1-1.5; the history of craniocerebral inflammation, swelling, trauma, operation history was excluded; the patients with mental illness and unable to cooperate with the examination were excluded.

## Methods

All patients underwent SWI, DTI, DKI and QSM sequence scanning on 3.0T MRI machine (Ingenia; Philips, best, the Netherlands), and the scan sequence parameters (Table 1).

**Table 1:** MRI scanning parameters

Name	TR (ms)	TE (ms)	Thickness (mm)		Number		FOV (mm×mm)		b (sec/mm^2^)
**SWI**	31	7.2/delta 6.2		2	130	230 × 189		—	
**DTI**	2773	85		2.5	48	224 × 224		0, 800	
**DKI**_**3b**_	3339	101		4	18	220 × 220		0, 1000, 2000	
**DKI**_**5b**_	3473	101		4	18	220 × 220		0, 500, 1000, 1500, 2000	
**QSM**	57	8/delta 6.3		2	18	220 × 220		—	

Note: SWI (susceptibility weighted imaging), DTI (diffusion tensor imaging), DKI (diffusion kurtosis imaging), QSM (quantitative susceptibility mapping), TR (time of repetition), TE (time of echo), FOV (field of view), b (diffusion sensitive factor)

The original image of DKI was exported in DICOM format. The images were preprocessed and classified. After post-processing with DKE (diffusion kurtosis estimator) software, mean kurtosis (MK), axial kurtosis (AK), radial kurtosis (RK), mean dispersion (MD) and fractional anisotropy (FA) were obtained. The parameters were imported into SPIN LITE software, which were read by two MRI doctors. The region of interest (ROI) was generated by hand animation, and the parameters MK, AK, RK, MD and FA were measured respectively.

After importing the scanned DTI images into Philips post-processing workstation, FA, ADC and neurotractography were obtained. The area of interest (ROI) was generated by hand animation and the parameters FA and ADC were measured.

The original image of QSM was exported in DICOM format, the data were preprocessed and classified, and the susceptibility parameter map was obtained by post-processing with Matlab software. The parameters were imported into image J software, which were read by two MRI doctors. The region of interest (ROI) was generated by hand animation and the parameter magnetic susceptibility was measured. Then the bilateral nigrosome-1 was observed.

The original SWI images were divided into amplitude map, phase map and angiogram. The images were read by two MRI doctors. The bilateral nigrosome-1 was observed by amplitude map.

The structure of bilateral igrosome-1 was judged on QSM image and SWI amplitude map. Based on the naked eye observation, it can be divided into three situations: clearly visible, suspected visible and invisible. According to the imaging diagnosis, the examinees were divided into three groups: ①Normal group: bilateral igrosome-1 was clearly visible; ② PD group: at least one side of igrosome-1 was not visible, including bilateral invisible, one side visible or suspected visible, the other side was not visible; ③Uncertain diagnosis group: the visibility of igrosome-1 was in other conditions, including bilateral igrosome-1 suspected visible, one side clearly visible, the other side suspected visible.

The DKI-MK value, DKI-AK value, DKI-RK value, DKI-RD value, DKI-FA value, DTI-FA value, DTI-ADC value and magnetic susceptibility value of five regions of interest (ROI) of bilateral substantia nigra, red nucleus, head of caudate nucleus, putamen and thalamus were selected. The igrosome-1 of bilateral substantia nigra in SWI and QSM images were observed and determined.

### Data analysis

SPSS 21.0 statistical software was used for analysis, and the observation value of each image was expressed as ($\bar X \pm S$). The process of data analysis is as follows:

(1) Age (*t* test) and gender (*χ*^*2*^test) were compared between PD group and HC group.

(2) The presence/absence of Nigerome-1 was observed by SWI and QSM respectively and was later used as imaging diagnosis of Parkinson’s disease. The imaging diagnosis was then compared with the clinical diagnosis, the gold standard in the study, in the following aspects: sensitivity, specificity, positive predictive value, negative predictive value and diagnostic coincidence rate.

(3) The *t* test (normal distribution) and nonparametric test (non normal distribution) were used to compare.

- the parameters (DKI, DTI, QSM) of each nucleus between the PD group and the HC group;
- the parameters of each nucleus between DKI_3b_ and DKI_5b_ images within the HC group;
- the parameters of each nucleus (DKI, DTI, QSM) between the PD group and the HC group.

(4) Pearson (bivariate normal distribution) / Spearman (bivariate incomplete normal distribution) correlation analysis was used to compare the correlation between the parameters of nuclei in PD group (DKI_3b_) and PD group (QSM). The correlation is considered statistically significant when *p* value is lower than 0.05.

## Results

### Clinical data of 40 examinees

There was no significant difference in age and gender between the two groups (*p* > 0.05) (Table 2).

**Table 2:** Clinical data of PD group and HC group

Variables	PD group	HC group	*p*
**Number**	20	20	-
**Age**	*66.50 ± 9.65*	*61.25 ± 6.87*	0.055*
**Gender (male / female)**	8/12	13/7	0.113**

Note: *Age comparison between PD group and HC group, **Gender comparison between PD group and HC group

### Visibility and diagnostic efficiency of SWI and QSM on Nigrosome-1 in substantia nigra

Based on SWI, 15 PD patients and 6 healthy volunteers were correctly diagnosed. The sensitivity, specificity, positive predictive value, negative predictive value and diagnostic coincidence rate of Nigrosome-1 for PD were 75.0%, 30.0%, 51.7%, 54.5%, and 52.5%, respectively. The positive likelihood ratio (+ LR) was 1.07, negative likelihood ratio (-LR) was 0.83 (Table 3).

**Table 3:** The relationship between the prevalence of PD group and HC group and the results of SWI

SWI	Gold Standard		Total
	PD group	HC group	
**PD group**	15	14	29
**HC group**	5	6	11
**Total**	20	20	40

**Fig. 1:**
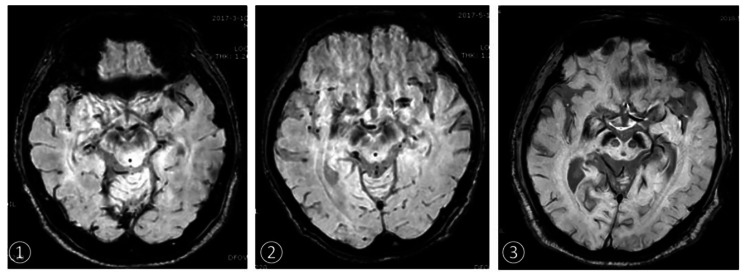
On the substantia nigra of SWI images ①HC group, female, 58 years old (bilateral Nigrosome-1 showed clearly) ②PD group, female, 63 years old, course of disease 1 year (right Nigrosome-1 is not clear, left Nigrosome-1 is clear) ③PD group, female, 65 years old, course of disease 5 years (bilateral Nigrosome-1 is not clear)

According to QSM, 19 PD patients and 11 healthy volunteers were diagnosed correctly. The sensitivity, specificity, positive predictive value and negative predictive value of one in the diagnosis of PD were 95.0%, 55.0%, 67.9%, 91.7% and 75.0%, respectively. The positive likelihood ratio (+ LR) was 2.11, negative likelihood ratio(-LR) was 0.09 (Table 4) (Fig. 1).

**Table 4:** The relationship between the prevalence of PD and HC and the results of QSM

QSM	Gold Standard		Total
	PD group	HC group	
**PD group**	19	9	28
**HC group**	1	11	12
**Total**	20	20	40

### Comparison of DKI_5b_ parameters between left and right sides of ROI position in HC group

There was no significant difference between the left and right sides of ROI **position** in healthy subjects (*p* > 0.05) (Table 5).

**Table 5:** The parameters of DKI5b in HC group were compared in ROI position

ROI position	parameter	L($\bar X \pm S$)		R($\bar X \pm S$)	*t/Z*	*p*
**Substantia nigra**	**MK**	*0.79 ± 0.23*	*0.84 ± 0.24*		-0.615	0.542
	**AK**	*0.59 ± 0.23*	*0.68 ± 0.24*		-1.244z	0.213
	**RK**	*0.89 ± 0.23*	*0.87 ± 0.30*		-0.352z	0.725
	**MD**	*0.37 ± 0.06*	*0.36 ± 0.06*		0.746	0.460
	**FA**	*0.21 ± 0.07*	*0.20 ± 0.04*		-0.108z	0.914
**Red nucleus**	**MK**	*0.76 ± 0.29*	*0.76 ± 0.32*		-0.076	0.940
	**AK**	*0.49 ± 0.24*	*0.57 ± 0.26*		-0.622z	0.534
	**RK**	*0.87 ± 0.33*	*0.90 ± 0.38*		-0.317	0.753
	**MD**	*0.39 ± 0.07*	*0.38 ± 0.06*		0.466	0.644
	**FA**	*0.19 ± 0.04*	*0.19 ± 0.04*		-0.287z	0.774
**Head of caudate nucleus**	**MK**	*0.60 ± 0.40*	*0.55 ± 0.38*		0.448	0.659
	**AK**	*0.54 ± 0.33*	*0.67 ± 0.40*		-1.079	0.287
	**RK**	*0.77 ± 0.45*	*0.74 ± 0.46*		0.238	0.815
	**MD**	*0.41 ± 0.07*	*0.44 ± 0.09*		-1.447	0.156
	**FA**	*0.09 ± 0.03*	*0.08 ± 0.02*		1.476	0.148
**Putamen**	**MK**	*0.45 ± 0.20*	*0.53 ± 0.14*		-1.354	0.184
	**AK**	*0.55 ± 0.21*	*0.62 ± 0.24*		-1.082z	0.279
	**RK**	*0.55 ± 0.20*	*0.66 ± 0.27*		-1.465	0.151
	**MD**	*0.41 ± 0.08*	*0.43 ± 0.13*		-0.108z	0.914
	**FA**	*0.10 ± 0.04*	*0.08 ± 0.02*		-1.136z	0.256
**Thalamus**	**MK**	*0.61 ± 0.28*	*0.60 ± 0.26*		0.156	0.877
	**AK**	*0.55 ± 0.17*	*0.52 ± 0.16*		0.673	0.505
	**RK**	*0.74 ± 0.27*	*0.73 ± 0.25*		0.025	0.980
	**MD**	*0.42 ± 0.08*	*0.43 ± 0.08*		-0.408	0.685
	**FA**	*0.14 ± 0.03*	*0.13 ± 0.02*		1.413	0.166

Note: z-rank sum test

### Comparison of DKI_3b_ parameters between left and right sides of ROI position in HC group

There was no significant difference between the left and right sides of ROI position in HC group (*p* > 0.05) (Table 6).

**Table 6:** The parameters of DKI3b in HC group were compared in ROI position

ROI position	Parameter	L($\bar X \pm S$)	R($\bar X \pm S$)	*t/Z*	*p*
**Substantia nigra**	**MK**	*0.74 ± 0.34*	*0.76 ± 0.30*	-0.245	0.808
	**AK**	*0.53 ± 0.25*	*0.59 ± 0.32*	-0.271z	0.787
	**RK**	*0.75 ± 0.36*	*0.93 ± 0.35*	-1.598	0.118
	**MD**	*0.37 ± 0.05*	*0.36 ± 0.05*	0.607	0.548
	**FA**	*0.21 ± 0.07*	*0.20 ± 0.04*	0.327	0.746
**Red nucleus**	**MK**	*0.67 ± 0.41*	*0.78 ± 0.36*	-0.839z	0.402
	**AK**	*0.54 ± 0.29*	*0.73 ± 0.40*	-1.717	0.094
	**RK**	*0.80 ± 0.34*	*0.78 ± 0.36*	-0.108z	0.914
	**MD**	*0.40 ± 0.07*	*0.40 ± 0.08*	0.158	0.875
	**FA**	*0.19 ± 0.05*	*0.19 ± 0.04*	0.352	0.726
**Head of caudate nucleus**	**MK**	*0.60 ± 0.39*	*0.58 ± 0.39*	0.186	0.854
	**AK**	*0.61 ± 0.35*	*0.55 ± 0.34*	0.513	0.611
	**RK**	*0.78 ± 0.45*	*0.74 ± 0.46*	0.223	0.824
	**MD**	*0.41 ± 0.08*	*0.43 ± 0.10*	-0.567	0.574
	**FA**	*0.10 ± 0.03*	*0.09 ± 0.03*	-0.568z	0.570
**Putamen**	**MK**	*0.63 ± 0.30*	*0.55 ± 0.26*	0.956	0.345
	**AK**	*0.59 ± 0.19*	*0.63 ± 0.22*	-0.501	0.619
	**RK**	*0.66 ± 0.27*	*0.65 ± 0.26*	0.105	0.917
	**MD**	*0.40 ± 0.10*	*0.42 ± 0.14*	-0.974	0.330
	**FA**	*0.09 ± 0.03*	*0.08 ± 0.03*	1.126	0.267
**Thalamus**	**MK**	*0.67 ± 0.23*	*0.64 ± 0.22*	0.373	0.711
	**AK**	*0.51 ± 0.21*	*0.62 ± 0.25*	-1.409	0.167
	**RK**	*0.71 ± 0.18*	*0.64 ± 0.20*	1.112	0.273
	**MD**	*0.41 ± 0.08*	*0.42 ± 0.07*	-0.356	0.723
	**FA**	*0.15 ± 0.04*	*0.13 ± 0.03*	1.322	0.194

Note: z-rank sum test

### Comparison of DKI parameters with different b values between left and right sides of ROI positions in HC group

There was no significant difference in ROI **position** between DKI_3b_ and DKI_5b_in HC group. (*p* > 0.05) (Table 7).

**Table 7:** Comparison of DKI3b and DKI5b parameters of ROI positions in HC group

ROI position	Parameter		DKI_3b_($\bar X \pm S$)	DKI_5b_($\bar X \pm S$)	*t/Z*	*p*
**Substantia nigra**	**MK**	*0.75 ± 0.23*		*0.82 ± 0.17*	-1.093	0.288
	**AK**	*0.56 ± 0.21*		*0.64 ± 0.21*	-1.008z	0.313
	**RK**	*0.84 ± 0.24*		*0.88 ± 0.22*	-0.606	0.552
	**MD**	*0.37 ± 0.05*		*0.36 ± 0.06*	0.446	0.661
	**FA**	*0.20 ± 0.05*		*0.20 ± 0.05*	0.042	0.967
**Red nucleus**	**MK**	*0.72 ± 0.31*		*0.76 ± 0.22*	-0.386	0.704
	**AK**	*0.63 ± 0.22*		*0.53 ± 0.19*	-1.120z	0.263
	**RK**	*0.79 ± 0.27*		*0.88 ± 0.22*	-1.139	0.269
	**MD**	*0.40 ± 0.07*		*0.39 ± 0.07*	1.086	0.291
	**FA**	*0.19 ± 0.04*		*0.19 ± 0.04*	-0.402	0.692
**Head of caudate nucleus**	**MK**	*0.58 ± 0.30*		*0.58 ± 0.31*	-0.822z	0.411
	**AK**	*0.58 ± 0.25*		*0.61 ± 0.27*	-0.331	0.745
	**RK**	*0.76 ± 0.35*		*0.76 ± 0.34*	-1.926z	0.054
	**MD**	*0.42 ± 0.08*		*0.42 ± 0.07*	-0.400	0.694
	**FA**	*0.09 ± 0.02*		*0.09 ± 0.02*	1.565	0.134
**Putamen**	**MK**	*0.59 ± 0.22*		*0.49 ± 0.13*	1.745	0.099
	**AK**	*0.61 ± 0.16*		*0.59 ± 0.19*	0.557	0.584
	**RK**	*0.65 ± 0.22*		*0.60 ± 0.18*	0.825	0.419
	**MD**	*0.41 ± 0.11*		*0.42 ± 0.10*	− .299z	0.765
	**FA**	*0.09 ± 0.03*		*0.09 ± 0.03*	-0.055	0.957
**Thalamus**	**MK**	*0.66 ± 0.20*		*0.60 ± 0.21*	0.839	0.412
	**AK**	*0.56 ± 0.20*		*0.53 ± 0.13*	0.562	0.581
	**RK**	*0.67 ± 0.12*		*0.74 ± 0.21*	-1.145	0.266
	**MD**	*0.42 ± 0.07*		*0.43 ± 0.08*	-0.433	0.670
	**FA**	*0.14 ± 0.03*		*0.14 ± 0.03*	0.175	0.863

Note: z-rank sum test

### Comparison of DKI_3b_ parameters between left and right sides of ROI in PD group

**Fig. 2:**
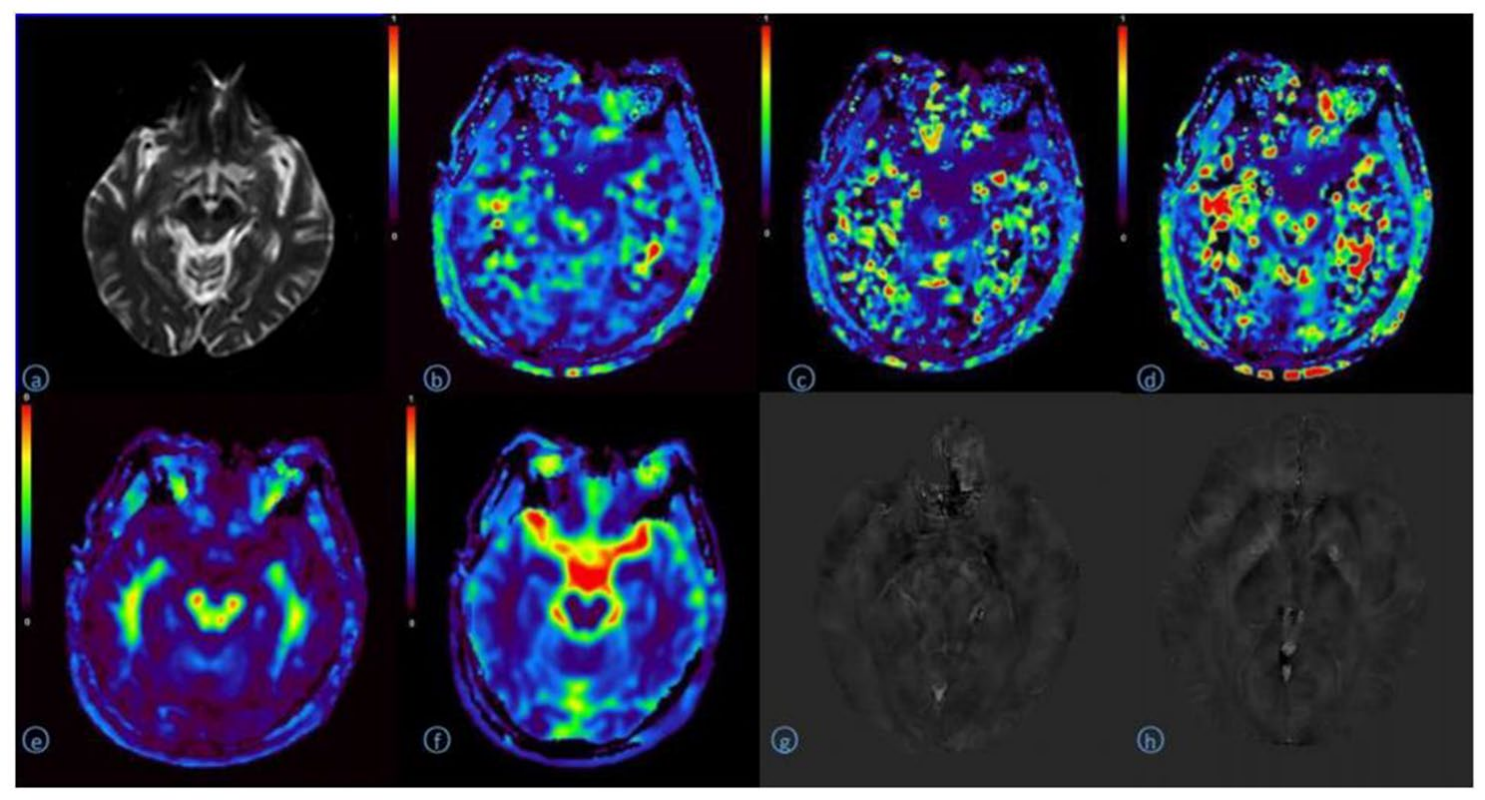
A 68 year old man with Parkinson’s disease A:B0 image of DKI original image (Substantia nigra, Red nucleus) B:DKI-MK pseudo color image (Substantia nigra 0.95, Red nucleus 0.88) C:DKI-AK pseudo color image(Substantia nigra 0.58, Red nucleus 0.46) d:DKI-RK pseudo color image(Substantia nigra 1.32, Red nucleus1.07) E:DKI-MD pseudo color image(Substantia nigra 0.52, Red nucleus 0.48) F:DKI-FA pseudo color image(Substantia nigra 0.23, Red nucleus 0.22) G:QSM image(Substantia nigra 0.20, red nucleus 0.19) h:QSM image(Head of caudate nucleus 0.06, Putamen 0.04, Thalamus 0.01)

There was no significant difference between the left and right sides of ROI in PD group (*p* > 0.05) (Table 8) (Fig. 2).

**Table 8:** Comparison of DKI3b parameters between left and right sides of ROI positions in PD group

ROI position	Parameter	L($\bar X \pm S$)	R($\bar X \pm S$)	*t/Z*	*p*
**Substantia nigra**	**MK**	*0.96 ± 0.30*	*0.93 ± 0.25*	0.926	0.366
	**AK**	*0.60 ± 0.20*	*0.52 ± 0.17*	-1.150z	0.250
	**RK**	*1.04 ± 0.49*	*0.90 ± 0.43*	0.955	0.346
	**MD**	*0.46 ± 0.08*	*0.44 ± 0.06*	-0.622z	0.534
	**FA**	*0.22 ± 0.08*	*0.21 ± 0.06*	-0.325	0.745
**Red nucleus**	**MK**	*0.68 ± 0.31*	*0.74 ± 0.30*	-0.613	0.543
	**AK**	*0.58 ± 0.26*	*0.52 ± 0.23*	-1.393	0.164
	**RK**	*0.90 ± 0.42*	*0.89 ± 0.41*	0.064	0.949
	**MD**	*0.44 ± 0.07*	*0.45 ± 0.08*	-0.392	0.695
	**FA**	*0.20 ± 0.08*	*0.19 ± 0.06*	-0.176z	0.860
**Head of caudate nucleus**	**MK**	*0.42 ± 0.28*	*0.47 ± 0.33*	-0.298z	0.766
	**AK**	*0.57 ± 0.35*	*0.52 ± 0.39*	-0.649z	0.516
	**RK**	*0.52 ± 0.32*	*0.59 ± 0.44*	-0.566	0.575
	**MD**	*0.46 ± 0.09*	*0.50 ± 0.12*	-0.974z	0.330
	**FA**	*0.10 ± 0.04*	*0.08 ± 0.02*	-1.272z	0.204
**Putamen**	**MK**	*0.58 ± 0.21*	*0.48 ± 0.27*	1.238	0.223
	**AK**	*0.56 ± 0.36*	*0.62 ± 0.34*	-0.487z	0.626
	**RK**	*0.70 ± 0.27*	*0.55 ± 0.31*	1.674	0.102
	**MD**	*0.46 ± 0.11*	*0.44 ± 0.10*	-0.730	0.465
	**FA**	*0.10 ± 0.04*	*0.10 ± 0.04*	-0.298z	0.766
**Thalamus**	**MK**	*0.52 ± 0.26*	*0.55 ± 0.31*	-0.325z	0.745
	**AK**	*0.54 ± 0.25*	*0.56 ± 0.29*	-0.054z	0.957
	**RK**	*0.74 ± 0.33*	*0.81 ± 0.36*	-0.575	0.569
	**MD**	*0.45 ± 0.10*	*0.45 ± 0.10*	-0.230z	0.818
	**FA**	*0.16 ± 0.07*	*0.15 ± 0.08*	-0.717	0.473

Note: z-rank sum test

### Comparison of ROI positions in DKI_3b_between PD group and HC group

**Fig. 3:**
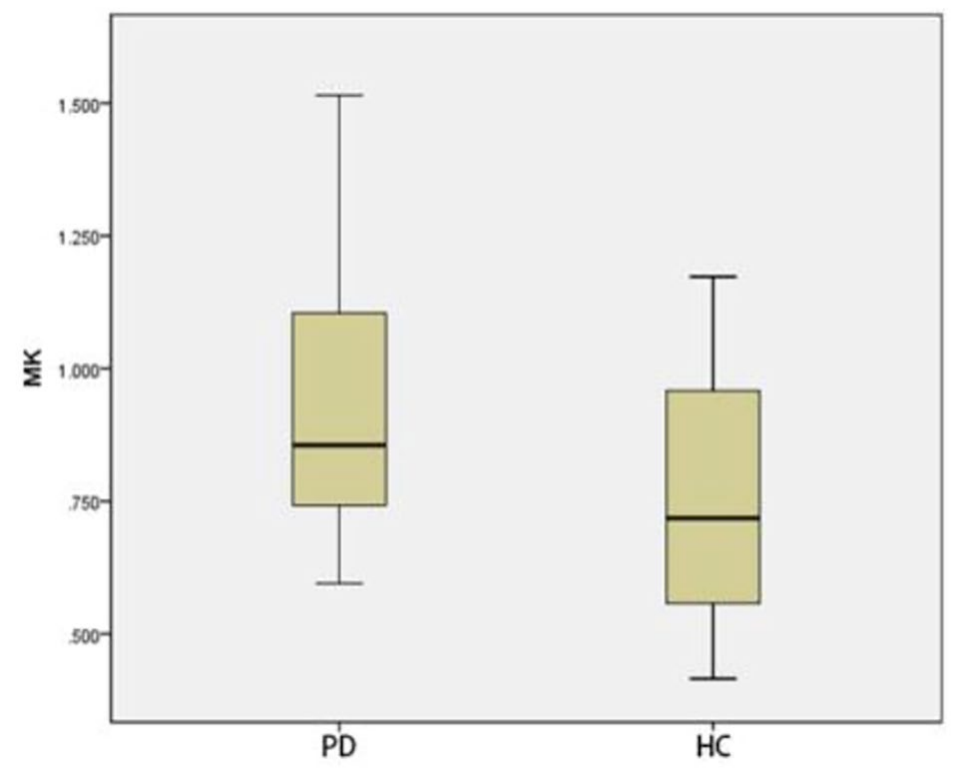
Two groups of MK box-plot of substantia nigra (*p* = 0.020)

**Fig. 4:**
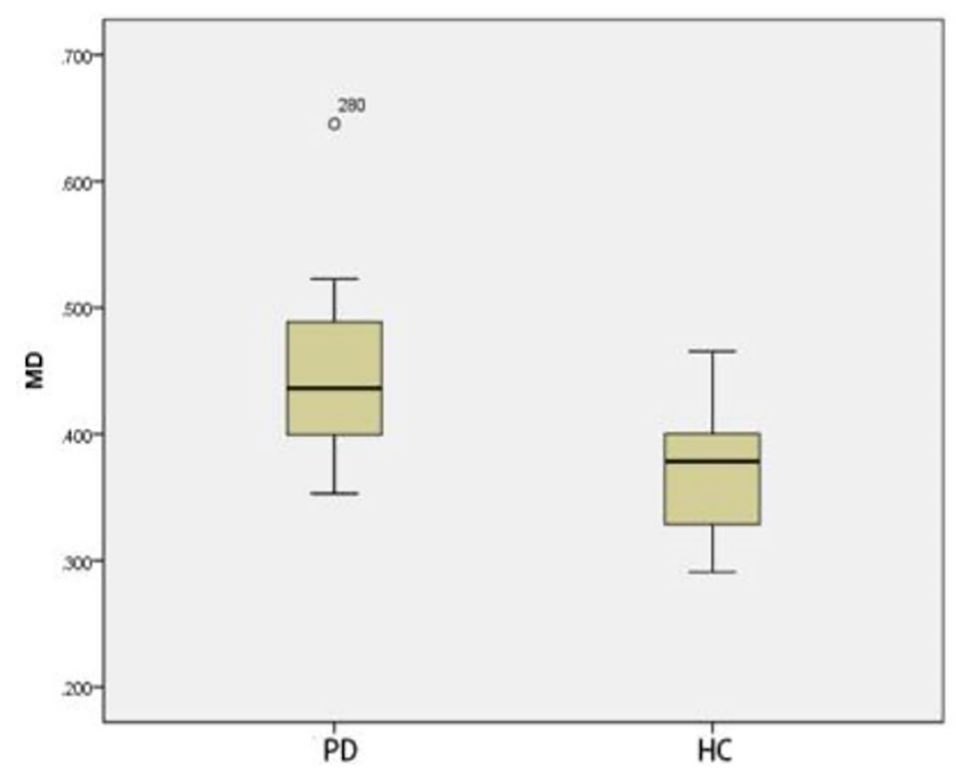
Two groups of MD box-plot of substantia nigra (*p* < 0.001)

**Fig. 5:**
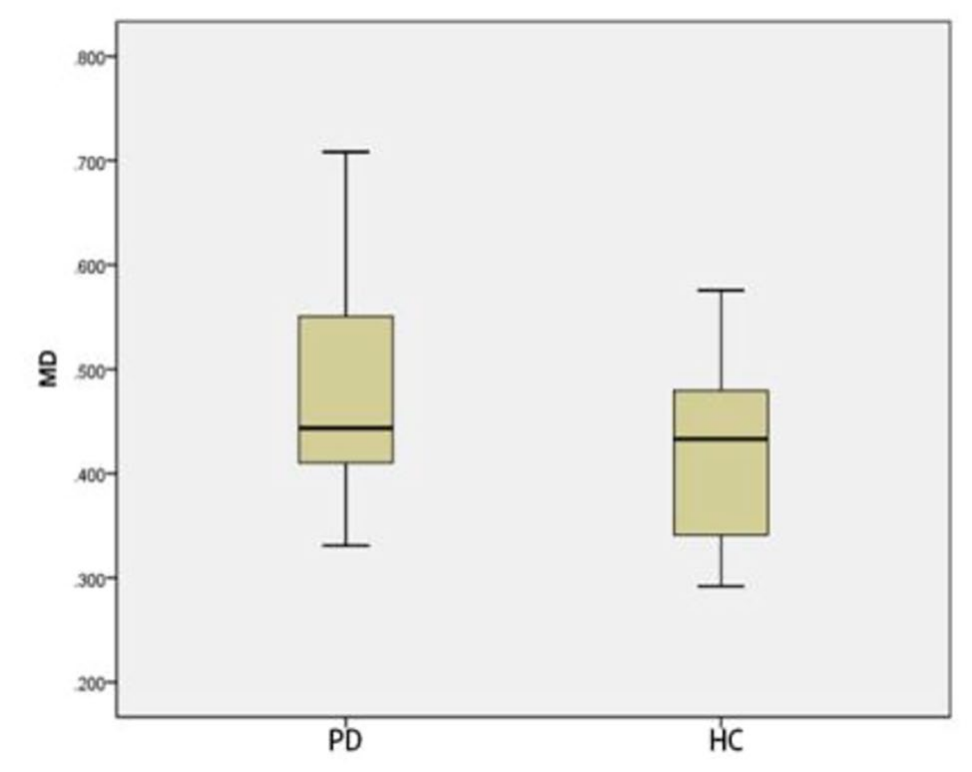
Two groups of MD box-plot of head of caudate nucleus (*p* = 0.043)

**Fig. 6:**
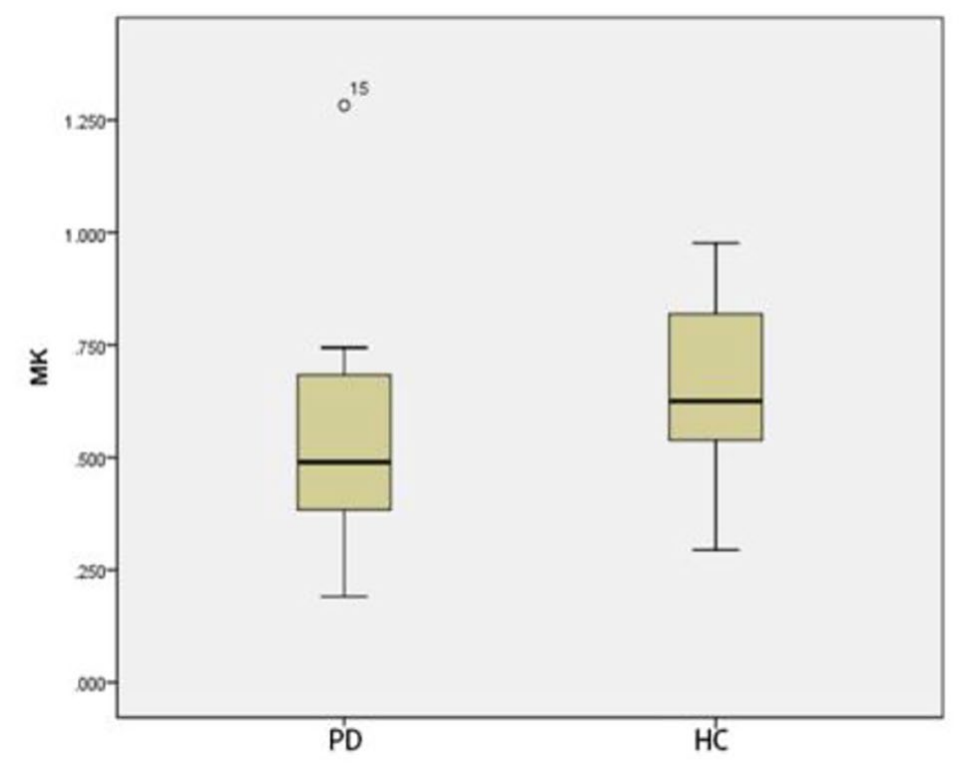
Two groups of MK box-plot of thalamus (*p* = 0.037)

Compared with that of HC group, The MK and MD in substantia nigra of PD group was significantly greater (*p* < 0.05) (Figs. 3 and 4). The MD of caudate nucleus head of PD group was significantly greater (*p* < 0.05) (Fig. 5). The MK of thalamus of PD group was significantly lower than that of the HC group (*p* < 0.05) (Fig. 6) (Table 9).

**Table 9:** Comparison of ROI positions in DKI3b between PD group and HC group

ROI position	Parameter		PD group ($\bar X \pm S$)	HC group ($\bar X \pm S$)	*t/Z*	*p*
**Substantia nigra**	**MK**	*0.94 ± 0.27*		*0.75 ± 0.23*	2.424	0.020
	**AK**	*0.56 ± 0.16*		*0.56 ± 0.21*	-0.460z	0.646
	**RK**	*0.97 ± 0.39*		*0.84 ± 0.24*	1.299	0.202
	**MD**	*0.45 ± 0.07*		*0.37 ± 0.05*	4.055	< 0.001
	**FA**	*0.22 ± 0.06*		*0.2 ± 0.05*	-0.189	0.850
**Red nucleus**	**MK**	*0.71 ± 0.23*		*0.72 ± 0.31*	-0.207	0.837
	**AK**	*0.55 ± 0.20*		*0.63 ± 0.22*	-1.407z	0.160
	**RK**	*0.90 ± 0.32*		*0.79 ± 0.27*	1.162	0.252
	**MD**	*0.45 ± 0.07*		*0.40 ± 0.07*	-1.758	0.079
	**FA**	*0.19 ± 0.06*		*0.19 ± 0.04*	-0.216	0.829
**Head of caudate nucleus**	**MK**	*0.44 ± 0.27*		*0.59 ± 0.30*	-1.704z	0.088
	**AK**	*0.54 ± 0.32*		*0.58 ± 0.25*	-1.082z	0.279
	**RK**	*0.55 ± 0.32*		*0.76 ± 0.35*	-1.839z	0.066
	**MD**	*0.48 ± 0.10*		*0.42 ± 0.08*	2.097	0.043
	**FA**	*0.09 ± 0.03*		*0.09 ± 0.02*	-1.109z	0.267
**Putamen**	**MK**	*0.53 ± 0.17*		*0.59 ± 0.21*	-1.066	0.293
	**AK**	*0.59 ± 0.32*		*0.61 ± 0.16*	-1.028z	0.304
	**RK**	*0.62 ± 0.18*		*0.65 ± 0.22*	-0.491	0.626
	**MD**	*0.45 ± 0.10*		*0.41 ± 0.11*	-1.488z	0.137
	**FA**	*0.10 ± 0.04*		*0.09 ± 0.03*	-0.893z	0.372
**Thalamus**	**MK**	*0.54 ± 0.24*		*0.66 ± 0.20*	-2.083	0.037
	**AK**	*0.55 ± 0.25*		*0.56 ± 0.20*	-0.239	0.812
	**RK**	*0.78 ± 0.28*		*0.67 ± 0.12*	1.483	0.146
	**MD**	*0.45 ± 0.10*		*0.42 ± 0.07*	-0.839z	0.402
	**FA**	*0.15 ± 0.07*		*0.14 ± 0.03*	-0.054z	0.957

Note: z-rank sum test

### Comparison of DTI parameters between left and right sides of ROI in HC group

There was no significant difference in DTI parameters between left and right sides of ROI in HC group. (*p* > 0.05) (Table 10).

**Table 10:** Comparison of DTI parameters between left and right sides of ROI positions in HC group

ROI position	Parameter		L($\bar X \pm S$)	R($\bar X \pm S$)	*t/Z*	*p*
**Substantia nigra**	**FA**	*0.40 ± 0.12*		*0.42 ± 0.08*	0.992	0.334
	**ADC**	*0.50 ± 0.10*		*0.49 ± 0.09*	0.084	0.934
**Red nucleus**	**FA**	*0.48 ± 0.10*		*0.49 ± 0.11*	0.380	0.708
	**ADC**	*0.47 ± 0.09*		*0.46 ± 0.09*	0.523z	0.601
**Head of caudate nucleus**	**FA**	*0.19 ± 0.04*		*0.18 ± 0.03*	0.475	0.640
	**ADC**	*0.66 ± 0.07*		*0.67 ± 0.05*	0.499z	0.618
**Putamen**	**FA**	*0.18 ± 0.05*		*0.16 ± 0.06*	1.858z	0.063
	**ADC**	*0.62 ± 0.04*		*0.65 ± 0.08*	1.220z	0.222
**Thalamus**	**FA**	*0.26 ± 0.03*		*0.26 ± 0.03*	0.322	0.751
	**ADC**	*0.67 ± 0.04*		*0.67 ± 0.04*	1.532	0.142

Note: z-rank sum test

### Comparison of DTI parameters between left and right sides of ROI in PD group

**Fig. 7:**
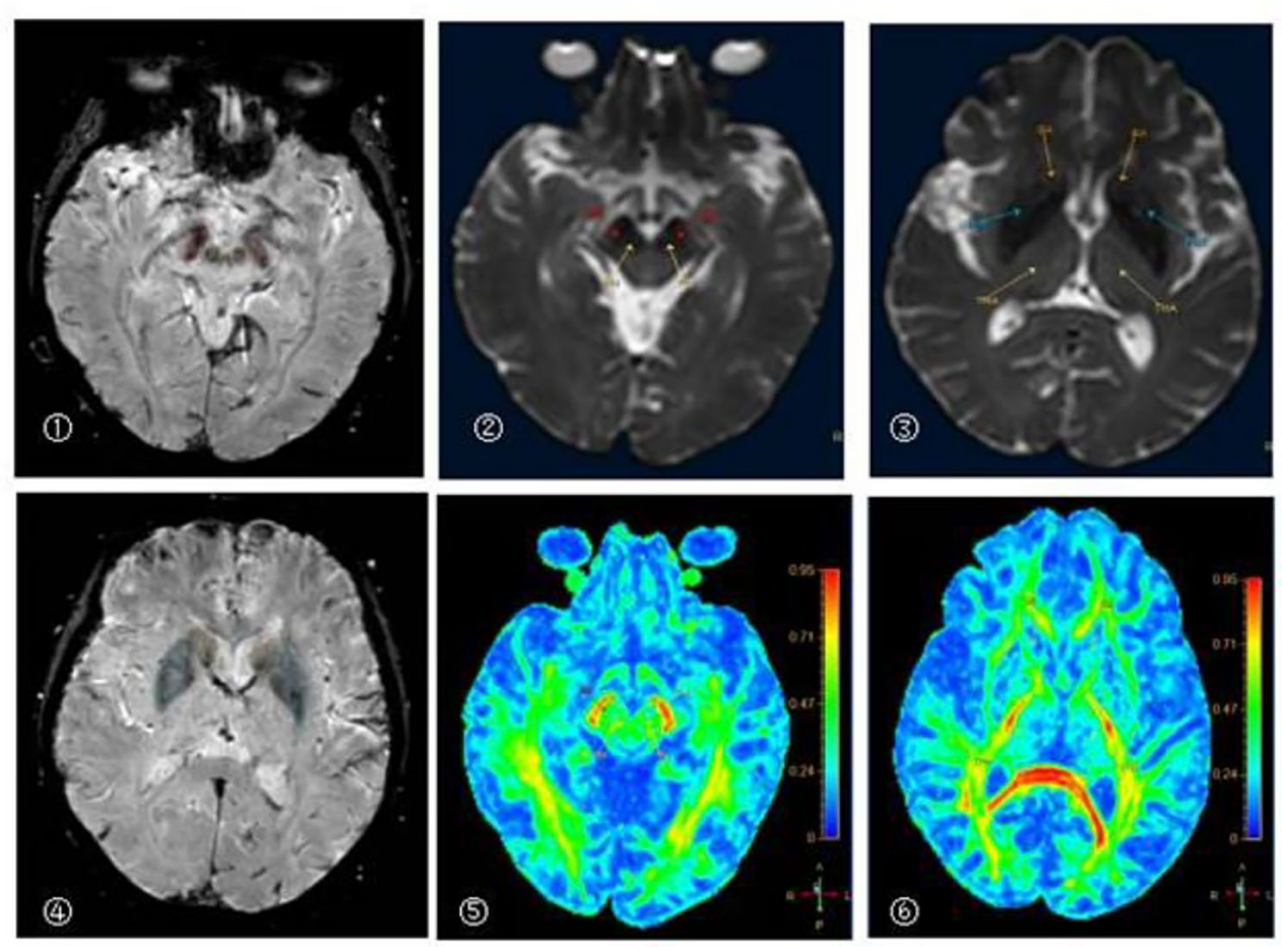
A 68 year old man with Parkinson’s disease ①②⑤The pseudo color images of SWI, B0 and DTI-FA in substantia nigra and red nucleus ③④⑥The pseudo color images of SWI, B0 and DTI-FA in head of caudate nucleus, putamen and thalamus

There was no significant difference in DTI parameters between left and right sides of ROI in PD group (*p* > 0.05) (Table 11) (Fig. 7).

**Table 11:** Comparison of DTI parameters between left and right sides of ROI positions in PD group

ROI position	Parameter	L($\bar X \pm S$)	R($\bar X \pm S$)	*t/Z*	*p*
**Substantia nigra**	**FA**	*0.39 ± 0.14*	*0.43 ± 0.07*	1.831z	0.067
	**ADC**	*0.53 ± 0.11*	*0.54 ± 0.08*	0.369	0.716
**Red nucleus**	**FA**	*0.46 ± 0.08*	*0.45 ± 0.07*	0.146	0.885
	**ADC**	*0.49 ± 0.05*	*0.49 ± 0.06*	0.370z	0.711
**Head of caudate nucleus**	**FA**	*0.20 ± 0.05*	*0.20 ± 0.04*	0.234	0.818
	**ADC**	*0.67 ± 0.06*	*0.68 ± 0.08*	0.969z	0.333
**Putamen**	**FA**	*0.17 ± 0.03*	*0.18 ± 0.04*	0.647z	0.518
	**ADC**	*0.64 ± 0.09*	*0.64 ± 0.08*	0.409z	0.682
**Thalamus**	**FA**	*0.28 ± 0.06*	*0.26 ± 0.04*	1.916	0.071
	**ADC**	*0.68 ± 0.06*	*0.70 ± 0.05*	2.046	0.055

Note: z-rank sum test

### Comparison of ROI positions in DTI parameters between PD group and HC group

There was no significant difference in DTI parameters between ROI positions of PD group and HC group (*p* > 0.05) (Table 12).

**Table 12:** Comparison of ROI positions in DTI parameters between PD group and HC group

ROI position	Parameter		HC group ($\bar X \pm S$)	PD group ($\bar X \pm S$)	*t/Z*	*p*
**Substantia nigra**	**FA**	*0.41 ± 0.09*		*0.41 ± 0.07*	0.019	0.985
	**ADC**	*0.50 ± 0.09*		*0.53 ± 0.08*	1.421z	0.155
**Red nucleus**	**FA**	*0.49 ± 0.09*		*0.45 ± 0.07*	1.287	0.206
	**ADC**	*0.47 ± 0.08*		*0.49 ± 0.05*	0.488z	0.626
**Head of caudate nucleus**	**FA**	*0.18 ± 0.02*		*0.20 ± 0.03*	1.952	0.060
	**ADC**	*0.66 ± 0.05*		*0.67 ± 0.06*	0.651z	0.515
**Putamen**	**FA**	*0.17 ± 0.05*		*0.18 ± 0.03*	0.291	0.773
	**ADC**	*0.64 ± 0.05*		*0.64 ± 0.08*	0.434z	0.665
**Thalamus**	**FA**	*0.26 ± 0.03*		*0.27 ± 0.05*	0.527	0.602
	**ADC**	*0.67 ± 0.04*		*0.69 ± 0.05*	1.470	0.150

Note: z-rank sum test

### Comparison of QSM parameters between left and right sides of ROI positions in HC group

There was no significant difference in QSM parameters between the left and right sides of ROI positions in HC group (*p* > 0.05) (Table 13).

**Table 13:** Comparison of QSM parameters between left and right sides of ROI positions in HC group

ROI position	L($\bar X \pm S$)	R($\bar X \pm S$)	*t/Z*	*p*
**Substantia nigra**	*0.11 ± 0.08*	*0.13 ± 0.08*	-1.759z	0.079
**Red nucleus**	*0.10 ± 0.09*	*0.11 ± 0.08*	-0.257z	0.797
**Head of caudate nucleus**	*0.01 ± 0.04*	*0.02 ± 0.05*	-0.835	0.409
**Putamen**	*0.03 ± 0.04*	*0.02 ± 0.06*	0.287	0.776
**Thalamus**	*0.01 ± 0.03*	*-0.01 ± 0.04*	1.701	0.097

Note: z-rank sum test

### Comparison of QSM parameters between left and right sides of ROI positions in PD group

There was no significant difference in QSM parameters between the left and right sides of ROI positions in PD group (*p* > 0.05) (Table 14)(Fig. 2).

**Table 14:** Comparison of QSM parameters between left and right sides of ROI positions in PD group

ROI position	L($\bar X \pm S$)	R($\bar X \pm S$)	*t/Z*	*p*
**Substantia nigra**	*0.19 ± 0.06*	*0.19 ± 0.05*	-0.129	0.899
**Red nucleus**	*0.18 ± 0.06*	*0.18 ± 0.08*	0.104	0.918
**Head of caudate nucleus**	*0.07 ± 0.02*	*0.05 ± 0.02*	1.905	0.073
**Putamen**	*0.05 ± 0.02*	*0.06 ± 0.02*	-0.391	0.700
**Thalamus**	*0.00 ± 0.03*	*0.01 ± 0.02*	-0.530z	0.596

Note: z-rank sum test

### Comparison of ROI positions in QSM parameters between PD group and HC group

**Fig. 8:**
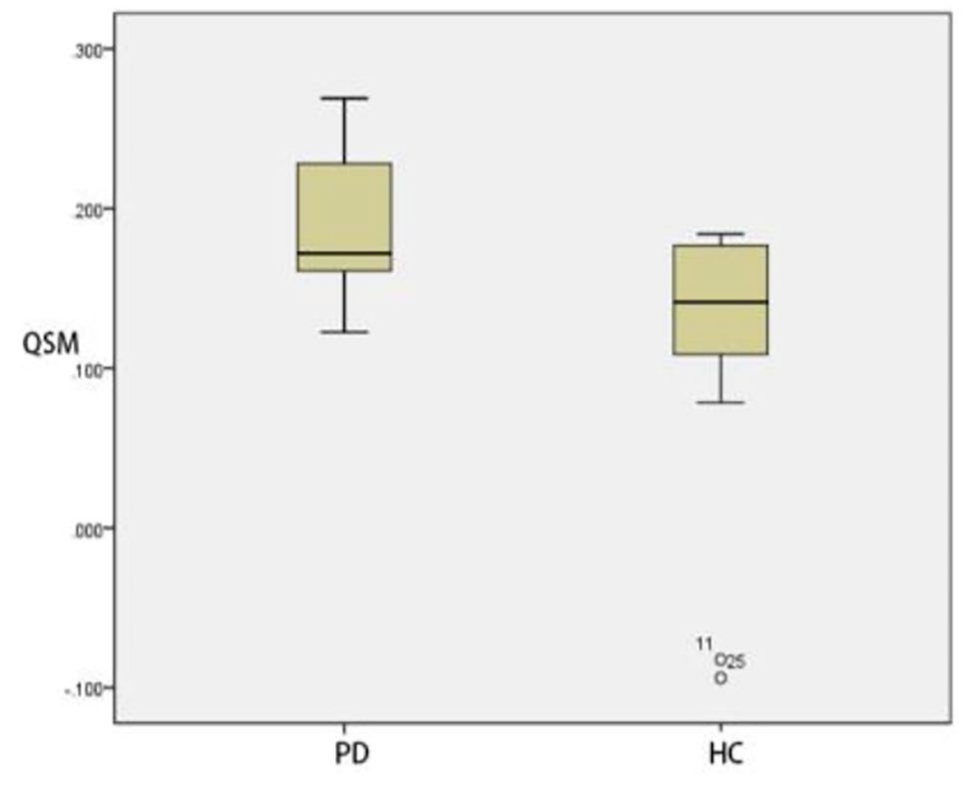
Two groups of QSM box-plot of substantia nigra (*p* = 0.028)

**Fig. 9:**
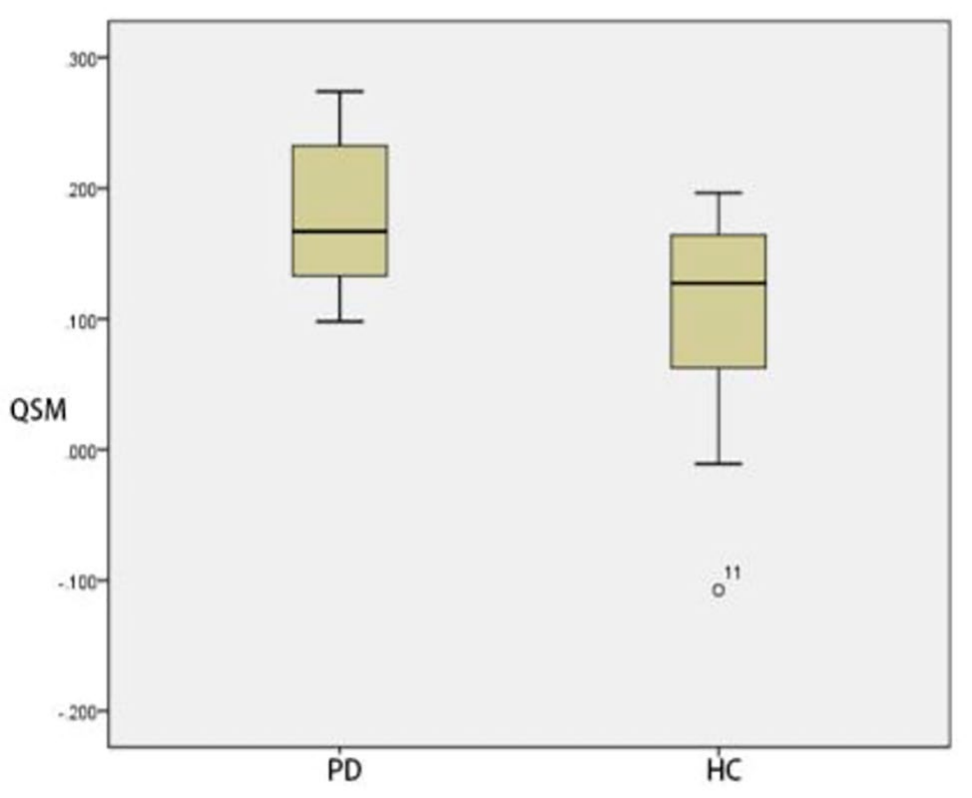
Two groups of QSM box-plot of red nucleus (*p* = 0.031)

**Fig. 10:**
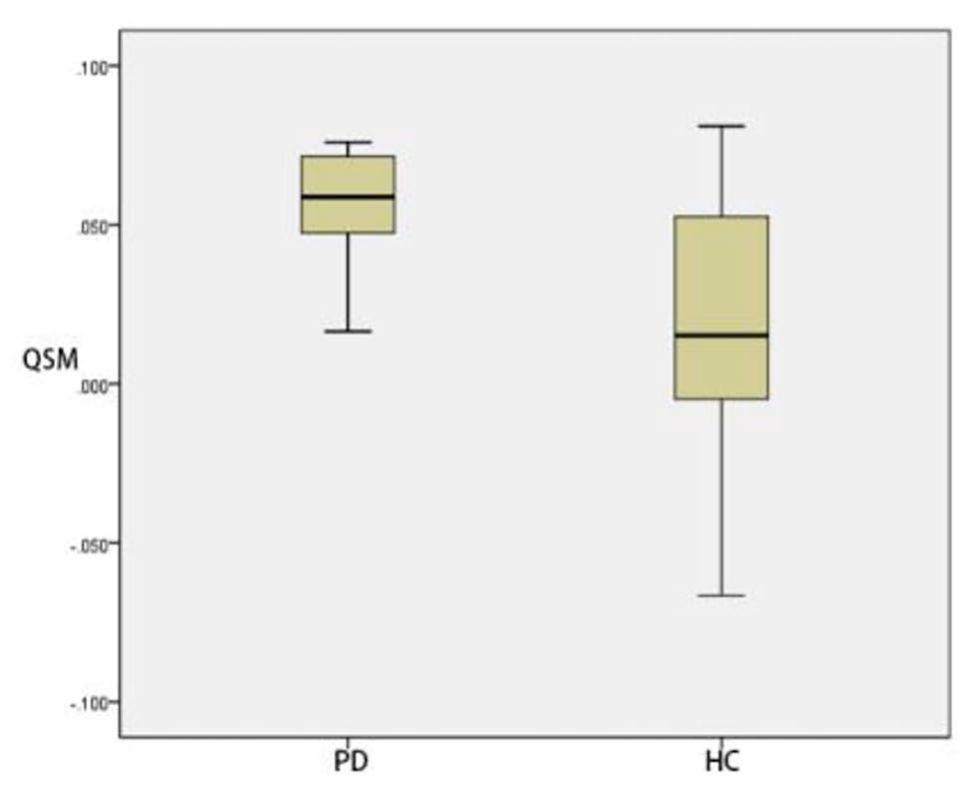
Two groups of QSM box-plot of head of caudate nucleus (*p* = 0.008)

**Fig. 11:**
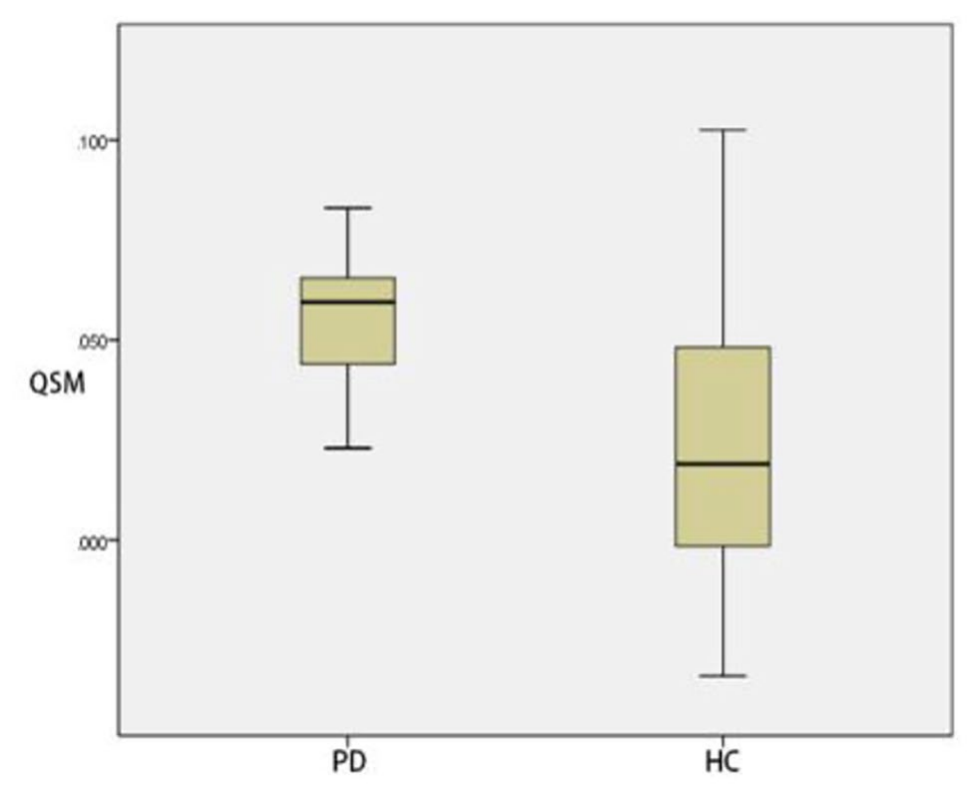
Two groups of QSM box-plot of putamen (*p* = 0.010)

In thalamus, there was no significant difference in QSM parameters between HC group and PD group (*p* > 0.05). By contrast, QSM of substantia nigra, red nucleus, head of caudate nucleus and putamen of PD group was significantly greater than those of HC group (*p* < 0.05) (Table 15) (Figs. 8, 9, 10 and 11).

**Table 15:** Comparison of ROI positions in QSM parameters between PD group and HC group

ROI position	HC group ($\bar X \pm S$)	PD group ($\bar X \pm S$)	*t/Z*	*p*
**Substantia nigra**	*0.19 ± 0.05*	*0.12 ± 0.08*	-2.200z	0.028
**Red nucleus**	*0.18 ± 0.06*	*0.11 ± 0.08*	-2.156z	0.031
**Head of caudate nucleus**	*0.06 ± 0.02*	*0.02 ± 0.04*	2.861	0.008
**Putamen**	*0.06 ± 0.02*	*0.02 ± 0.03*	2.762	0.010
**Thalamus**	*0.00 ± 0.01*	*0.00 ± 0.03*	0.381	0.706

Note: z-rank sum test

### Correlation analysis between QSM parameters and DKI parameters of ROI

There was no significant difference between QSM parameters and DKI parameters of ROI (*p* > 0.05) (Table 16).

**Table 16:** Correlation analysis between QSM parameters and DKI parameters of ROI

ROI	Substantia nigra	Red nucleus	Head of caudate nucleus	Putamen	Thalamus
**MK**	r = 0.090 P = 0.970	r = 0.050 P = 0.835	r=-0.226 P = 0.339	r=-0.212 P = 0.370	r = 0.126 P = 0.596
**AK**	r = 0.044 P = 0.855	r=-0.256 P = 0.277	r=-0.426 P = 0.061	r = 0.063 P = 0.791	r = 0.410 P = 0.073
**RK**	r=-0.239 P = 0.310	r = 0.158 P = 0.506	r=-0.237 P = 0.315	r=-0.213 P = 0.366	r=-0.077 P = 0.748
**MD**	r = 0.205 P = 0.387	r = 0.197 P = 0.405	r=-0.358 P = 0.121	r = 0.176 P = 0.458	r = 0.054 P = 0.821
**FA**	r=-0.285 P = 0.223	r=-0.153 P = 0.519	r=-0.385 P = 0.094	r=-0.174 P = 0.464	r=-0.167 P = 0.482

### The ROC curve of DKI parameters at each ROI position

**Fig. 12:**
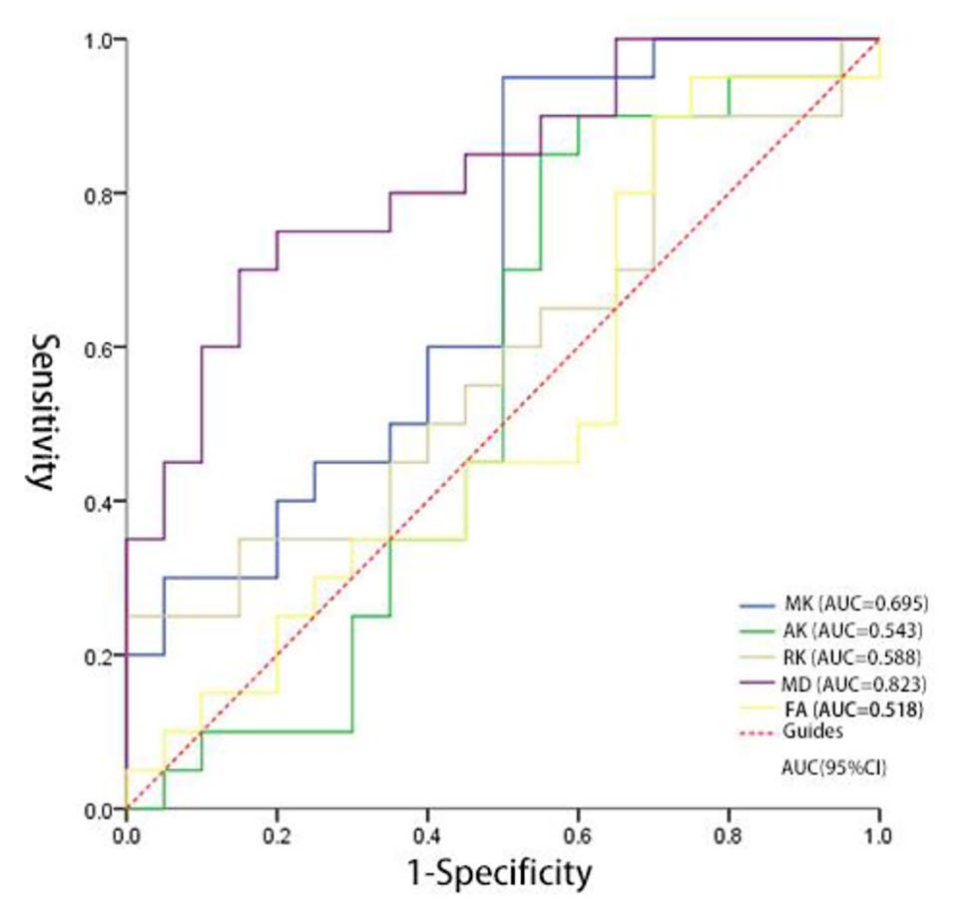
The ROC curves of HC group and PD group of the parameters of substantia nigra position

The maximum area under the ROC curve (AUC) of MD value was 0.823, the sensitivity was 70.0%, the specificity was 85.0%, and the diagnostic threshold was 0.414; the area under the ROC curve (AUC) of MK value in substantia nigra was 0.695, the sensitivity was 95.0%, the specificity was 50.0%, and the diagnostic threshold was 0.667. Both were statistically significant (*p* < 0.05) (Table 17) (Fig. 12).

**Table 17:** ROC curve of DKI parameters at each ROI position

ROI position	Parameters		Sensitivity (%)	Specificity (%)	Yoden index	Threshold		AUC(95%CI)	*p*
**Substantia nigra**		**MK**	95.0	50.0	0.450	0.667	0.695(0.530,0.860)		0.035
		**AK**	85.0	45.0	0.300	0.408	0.543(0.354,0.731)		0.646
		**RK**	25.0	100.0	0.250	1.242	0.588(0.408,0.767)		0.344
		**MD**	70.0	85.0	0.550	0.414	0.823(0.695,0.950)		< 0.001
		**FA**	90.0	30.0	0.200	0.167	0.518(0.333,0.702)		0.850
**Red nucleus**		**MK**	95.0	25.0	0.200	0.424	0.513(0.327,0.698)		0.892
		**AK**	5.0	95.0	0.000	1.090	0.370(0.195,0.545)		0.160
		**RK**	25.0	95.0	0.200	1.225	0.583(0.403,0.762)		0.372
		**MD**	90.0	55.0	0.450	0.397	0.663(0.488,0.837)		0.079
		**FA**	50.0	60.0	0.100	0.183	0.480(0.297,0.663)		0.829
**Head of caudate nucleus**		**MK**	5.0	100.0	0.050	1.218	0.343(0.169,0.516)		0.088
		**AK**	100.0	10.0	0.100	0.112	0.400(0.218,0.582)		0.279
		**RK**	5.0	100.0	0.050	1.367	0.330(0.161,0.499)		0.066
		**MD**	95.0	35.0	0.300	0.367	0.648(0.475,0.820)		0.110
		**FA**	25.0	80.0	0.050	0.112	0.398(0.218,0.577)		0.267
**Putamen**		**MK**	95.0	15.0	0.100	0.337	0.380(0.199,0.561)		0.194
		**AK**	10.0	100.0	0.100	0.994	0.405(0.225,0.585)		0.304
		**RK**	45.0	70.0	0.150	0.645	0.475(0.291,0.659)		0.787
		**MD**	95.0	35.0	0.300	0.355	0.638(0.463,0.812)		0.137
		**FA**	100.0	20.0	0.200	0.059	0.583(0.403,0.762)		0.372
**Thalamus**		**MK**	5.0	100.0	0.050	1.130	0.308(0.140,0.475)		0.037
		**AK**	15.0	95.0	0.100	0.793	0.453(0.271,0.634)		0.607
		**RK**	55.0	85.0	0.400	0.781	0.638(0.455,0.820)		0.137
		**MD**	95.0	35.0	0.300	0.354	0.578(0.394,0.761)		0.402
		**FA**	30.0	85.0	0.150	0.171	0.505(0.322,0.688)		0.957

Note: z-rank sum test

## Discussion

The main pathological changes of Parkinson’s disease are the degeneration of dopaminergic neurons in substantia nigra of midbrain and the presence of fiber eosinophilic inclusion bodies, namely Lewy bodies, which are mainly composed of alpha synuclein and ubiquitin [13]. Moreover, recent studies have also suggested that the disorder of iron metabolism in the brain may lead to neurodegenerative diseases such as Parkinson’s disease. The iron content in substantia nigra of patients with Parkinson’s disease increases significantly, and with the aggravation of the disease, the iron deposition increases [14]. Although the biological mechanism of abnormal iron metabolism in PD patients is not clear, it is certain that Parkinson’s disease is closely related to the imbalance of iron absorption, storage, transport and release, which may be the main factor leading to the loss of dopaminergic neurons and abnormal aggregation of α-synuclein.

There was no significant difference between the left and right sides of gray matter nuclei in 40 examinees. It showed that the degree of disease change and the dominance of left and right hands in patients with early Parkinson’s disease would not significantly affect the results of the study.

DKI is an extension of diffusion tensor imaging (DTI). It is a practical clinical technique for quantifying the diffusion of non normal water molecules and exploring the microstructure of biological tissues. The application direction of diffusion sensitive gradient field in DKI technology needs at least 15, and can be increased to 30 gradient field directions. The number of b values can be 3 or 5 [15]. In this study, by plotting and measuring the DKI parameters with different b values in the deep nuclei of normal human brain, five regions of interest were selected, namely, the substantia nigra, the red nucleus, the head of caudate nucleus, putamen and thalamus. After data analysis, it was found that there was no statistical difference between DKI_3b_ scanning and DKI_5b_ scanning. Therefore, it can be considered that the reduction of b value will not affect the image and data processing Therefore, DKI_3b_ scan can be used instead of DKI_5b_ scan in clinical practice, which can greatly shorten the scanning time and reduce the occurrence of events such as Parkinson’s disease due to the long scanning time. This adaption may provide the basis for the new clinical application of DKI technology in the future.

The main parameters of DKI include mean kurtosis (MK), axial kurtosis (AK), radial kurtosis (RK) and kurtosis anisotropy (KA). Scanning DKI sequence can also obtain DTI related parameters, including fractional anisotropy (FA), mean diffusion (MD), axial diffusion tensor (AD), and vertical diffusion tensor (RD). The DKI related parameters have higher sensitivity and specificity than DTI related parameters in evaluating the changes of microstructure of deep gray matter nuclei. Moreover, the parameter maps of DKI play an important clinical application value in the evaluation of disease evolution and process, therapeutic effect and prognosis follow-up.

DKI-MK is a parameter reflecting the complexity of brain microstructure. It takes the mean value of different b value directions in the same direction gradient direction. The size of MK depends on the structural complexity of the organization within the ROI range. In normal human brain tissue, the complexity of different parts is different, so MK is also different. The loss of dopaminergic neurons, oxidative stress, neuritis and other factors in PD patients can cause changes in the complexity of different parts. In this study, the increase of MK in substantia nigra in this study may indicate that early inflammation leads to a large number of glial cells and cytokine activation, which is more than the loss of dopaminergic neurons, which may lead to the increase of local complexity, which is consistent with the report that early neuroinflammation can effectively alleviate the degeneration of dopaminergic neurons [16]. However, it is different from the conclusion of some researchers, which may be related to the duration of disease and the severity of disease, which may lead to the increase of neuron damage. There are subthalamic nuclei (STN) in the thalamus. Due to the excessive loss of dopaminergic cells in the substantia nigra compacta of PD, the number of cells projecting to the striatum is reduced, which can lead to over activation of STN pathway and inhibit the cerebral cortex [17]. Excessive activation of STN may aggravate the damage of local tissues, and may lead to the decrease of MK, which is consistent with the conclusion of this paper.

Although MK does not depend on the spatial orientation of organizational structure, it ignores the directionality of diffusion movement [18]. AK and RK make up for the deficiency of MK, which can reflect the value of dispersion kurtosis along the fiber bundle direction and perpendicular to the fiber bundle direction respectively. It was found that the RK of the gray matter nuclei in both HC and PD groups was significantly greater than that of AK, indicating that the dispersion of water molecules with non normal distribution in the direction perpendicular to the fiber bundle was more significant. It is possible that the AK is smaller because the water molecules in the axial direction are relatively free; because the diffusion of water molecules is limited due to the blockage of myelin sheath, the RK is larger [19].

MD reflects the overall diffusion level and resistance of water molecules. In this study, the substantia nigra and the head of caudate nucleus in PD patients were increased, which indicated that the loss and degeneration of neurons and the loose structure between tissues might lead to the faster diffusion of water molecules. In this study, the MK value of substantia nigra in PD patients increased, and the MD value also increased. The possible reason is that although inflammatory factors and other results lead to the increase of local complexity, it should be lower than the degree of normal brain tissue relationship, so the increase of MD value can also exist. FA value reflects the non-uniformity of diffusion direction and velocity of water molecules in tissues. There is no statistical difference in DKI-FA value and DTI-FA value in all parts of this study, which is different from the conclusion that FA in substantia nigra of PD patients is significantly decreased [20]. It may be due to the early stage of PD patients with no obvious pathological changes, or it may be caused by the loss of dopaminergic neurons, iron deposition, scanning parameters and other confounding factors.

Based on the presence or absence of SWI and QSM in the diagnosis of substantia nigra swallow tail sign, QSM has higher diagnostic efficiency and higher negative predictive rate, which is similar to the research results of He Naying and Xu Hongmin [21]. QSM technology is based on the needs of clinical research, Yi Wang research group of Cornell University proposed on the basis of susceptibility weighted imaging (SWI). It is a technique for quantitative measurement of tissue magnetization characteristics, which can be applied to neurological diseases based on abnormal iron content [22]. Parkinson’s disease is associated with the loss of dopaminergic cells and excessive iron deposition in nuclei such as substantia nigra. This study showed that compared with HC group, the magnetic susceptibility of substantia nigra, red nucleus, caudate nucleus and putamen in PD group were increased, which indicated that there was excessive iron deposition in patients with early Parkinson’s disease.

There was no significant correlation between the magnetic susceptibility of gray matter nuclei and the corresponding DKI parameters, indicating that the abnormal iron deposition could not completely reflect the changes of the microstructure of gray matter nuclei in Parkinson’s disease patients. The abnormal iron deposition may be only one aspect of it, and other factors should be considered together, such as the loss of a large number of dopaminergic neurons, microglia and astrocytes cell activation.

The prevalence of Parkinson’s disease is increasing year by year, and the clinical diagnosis lacks objectivity. This study uses SWI, QSM, DTI and DKI techniques to provide multiple data for analysis. According to the changes in the structure of substantia nigra, putamen and dorsal thalamus of PD patients, it is possible to find the changes in the fine anatomical structure and pathological changes of the gray matter nuclei in the brain, which will help improve the accuracy of PD diagnosis and provide objective basis for clinical diagnosis. The study was a non-large sample experiment with relatively few samples, which has certain limitations. The image post-processing was tedious, and there may be some errors in manual measurement. It is expected that in the future research, on the basis of increasing the number of samples, the research methods of this paper will be used to verify whether the results obtained are consistent.

This current study is limited by the relatively small sample size. As a result, future investigations into the accuracy of MRI in diagnosing PD and objective imaging indicators for early clinical diagnosis and treatment would be recommended. Future studies might provide more definitive evidence by expanding the sample size and refining the grouping of PD cases. Future studies might also deep-dive into the effectiveness of medication by comparing PD cases taking medication with those not taking medication and evaluating the treatment efficacy of PD cases taking medication.

## Data Availability

The datasets used and/or analysed during the current study are available from the corresponding author on reasonable request.
